# Probing magnetic orbitals and Berry curvature with circular dichroism in resonant inelastic X-ray scattering

**DOI:** 10.1038/s41535-023-00538-x

**Published:** 2023-01-19

**Authors:** Michael Schüler, Thorsten Schmitt, Philipp Werner

**Affiliations:** 1 Condensed Matter Theory Group, Paul Scherrer Institute, CH-5232 Villigen PSI, Switzerland; 2 Laboratory for Materials Simulations, Paul Scherrer Institute, CH-5232 Villigen PSI, Switzerland; 3 Department of Physics, University of Fribourg, 1700 Fribourg, Switzerland; 4 Photon Science Division, Swiss Light Source, Paul Scherrer Institute, CH-5232 Villigen PSI, Switzerland

**Keywords:** Electronic properties and materials, Topological matter

## Abstract

Resonant inelastic X-ray scattering (RIXS) can probe localized excitations at selected atoms in materials, including particle-hole transitions between the valence and conduction bands. These transitions are governed by fundamental properties of the corresponding Bloch wave functions, including orbital and magnetic degrees of freedom, and quantum geometric properties such as the Berry curvature. In particular, orbital angular momentum (OAM), which is closely linked to the Berry curvature, can exhibit a nontrivial momentum dependence. We demonstrate how information on such OAM textures can be extracted from the circular dichroism in RIXS. Based on accurate modeling with a first-principles treatment of the key ingredient—the light–matter interaction—we simulate dichroic RIXS spectra for the prototypical transition-metal dichalcogenide MoSe_2_ and the two-dimensional topological insulator 1T^′^-MoS_2_. Guided by an intuitive picture of the optical selection rules, we discuss how the momentum-dependent OAM manifests itself in the dichroic RIXS signal if one controls the momentum transfer. Our calculations are performed for typical experimental geometries and parameter regimes, and demonstrate the possibility of observing the predicted circular dichroism in forthcoming experiments. Thus, our work establishes a new avenue for observing Berry curvature and topological states in quantum materials.

## Introduction

The microscopic quantum geometry of Bloch electrons is one of the most remarkable properties of quantum materials, giving rise to fascinating macroscopic effects. Notable examples are topological insulators and superconductors^1,2^, where the integral over the Berry curvature yields an integer—the Chern number—that is fundamentally connected to edge modes and transport properties. More generally, the momentum-resolved Berry curvature plays a fundamental role in the optical properties of solids, including intriguing effects in high-harmonic generation^3,4^, the valley Hall effect^5^, and nonlinear Hall effect^6,7^.

Measuring the Berry curvature texture has proven difficult. The most promising route up to now is angle-resolved photoemission spectroscopy (ARPES)^8,9^ with circularly polarized photons. For example, the circular dichroism (CD) provides insights into the chirality in graphene^10^ and topological insulators^11^ and into the Berry curvature in two-dimensional materials^12–15^ and in Weyl semimetals^16^. Circular photons couple to the texture of the orbital angular momentum (OAM) of the Bloch states^17,18^, which, in turn, is closely connected to the Berry curvature^15,19^, although the precise relation can be complicated^16,20^. The connection between the OAM texture and the measured CD is governed by the photoemission matrix elements, which depend on the details of the final states^21,22^, the specifics of the experimental geometry^23^, mixing of different orbital channels^24^, and the short escape depth of the photoelectrons^25^. Especially for bulk materials, the link between the CD signal and OAM is obscured by these complications, rendering the extraction of Berry curvature a difficult task.

One of the most powerful techniques for revealing the magnetic properties of materials—including OAM—is resonant inelastic X-ray scattering (RIXS)^26^. This technique provides access to excitations in various degrees of freedom, including magnetic excitations such as magnons, as well as orbital excitations^27^. Controlling the polarization of the incoming X-ray photons allows to select or enhance certain excitations as governed by the selection rules. For *d* electron systems, this selectivity has been exploited to maximize spin-flip excitations^28^ to map out the magnetic degrees of freedom^29^. The quantum geometry of the Bloch states can impose further restrictions on the final states^30^. Based on this idea, ref. ^31^ has demonstrated how the chirality in Weyl semimetals leads to linear dichroism in RIXS. Thanks to recent advances in measuring RIXS with circularly polarized photons, it becomes possible to reveal OAM and thus measure local magnetic orbital excitations. Transitions between magnetic orbitals are a direct fingerprint of OAM localized at the targeted atoms. Hence, CD in RIXS (CD-RIXS) provides an unprecedented probe for the chirality of the electronic wave function and the breaking of time-reversal symmetry. CD-RIXS is conceptually similar to X-ray magnetic CD (XMCD) in X-ray absorption, and it has been used to study magnetic materials^32–36^, spin-flip processes^37^, or anti-ferromagnetic orbital order^38^. However, the link between the local OAM texture and CD-RIXS has not been explored.

Because Berry curvature is typically tied to local OAM—especially in transition-metal dichalcogenides (TMCDs), which are a prominent class of two-dimensional materials^39–41^—CD-RIXS can reveal quantum geometric information beyond basic magnetic properties. This novel aspect is the focus of this work. We show how CD-RIXS provides a fingerprint of magnetic orbital excitations in paradigmatic TMDCs. Exploiting the momentum resolution of RIXS, we explore how the *momentum-dependent* OAM texture manifests itself in the CD-RIXS signal, using accurate modeling with first-principle input. We focus on monolayer MoSe_2_ to demonstrate how the valley-dependent OAM can be mapped out, and on 1T^′^-MoS_2_, which is a representative of the class of intensively studied two-dimensional topological insulators^42–45^, in which Berry curvature and an associated dipole can be induced and tuned by applying an out-of-plane electric field^6^. We discuss how this intriguing scenario can be observed with CD-RIXS, underlining the power of CD-RIXS as a novel probe for quantum materials.

## Results

**Fig. 1:**
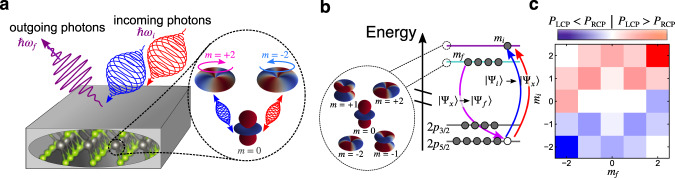
Sketch of the setup and dichroic selection rules. **a** We investigate resonant inelastic X-ray scattering (RIXS) with circularly polarized photons (with energy ℏ*ω*_*i*_) impinging on a sample, while scattered photons (with energy ℏ*ω*_*f*_) are detected without resolving their polarization. This setup is sensitive to local magnetic orbital excitations, which are controlled by the circular polarization of the incoming light. **b** Sketch of *d*-*d* excitations in the RIXS process: upon absorbing a circularly polarized photon, an electron is promoted from the 2*p* core levels to an unoccupied state in the *d* manifold with magnetic quantum number *m*_*i*_. A filled *d* orbital (quantum number *m*_*f*_) relaxes and fills the core hole. Effectively, this corresponds to an excitation within the *d* orbital space. **c** Circular dichroism of the *d*-*d* transition probability *P* resolved with respect to the magnetic quantum number of the *d* orbitals.

In the RIXS process, a core electron is promoted to the unoccupied bands upon absorbing an X-ray photon with energy ℏ*ω*_*i*_. The core hole is filled by a relaxing valence electron which emits an outgoing photon with lower energy ℏ*ω*_*f*_, such that the system absorbs the energy ℏΔ*ω* = ℏ*ω*_*i*_ − ℏ*ω*_*f*_ (see illustration in Fig. 1). The cross-section of this process is described theoretically by the Kramers–Heisenberg formula

1 $$I({\omega }_{i},{{{{\bf{q}}}}}_{i},{\omega }_{f},{{{{\bf{q}}}}}_{f})=\mathop{\sum}\limits_{f}{\left\vert {A}_{fi}({{{{\boldsymbol{\omega }}}}}_{i},{{{{\bf{q}}}}}_{i},{{{{\bf{q}}}}}_{f})\right\vert }^{2}\delta ({E}_{i}+\Delta \omega -{E}_{f}).$$

We use atomic units (a.u.) here and in what follows. The incoming (outgoing) X-ray photon carries the momentum **q**_*i*_ (**q**_*f*_). Thus the total momentum **q** = **q**_*f*_ − **q**_*i*_ is transfered to the system. Controlling the momentum transfer **q** via the experimental geometry provides momentum-resolved information, and allows to map out the dispersion of fundamental quasi-particle excitations such as phonons, magnons, and orbital excitations.

The RIXS signal (1) is determined by the RIXS amplitude

2 $${A}_{fi}({{{{\boldsymbol{\omega }}}}}_{i},{{{{\bf{q}}}}}_{i},{{{{\bf{q}}}}}_{f})=\mathop{\sum}\limits_{x}\frac{\langle {\Psi }_{f}| {\hat{\Delta }}_{f}^{{\dagger} }| {\Psi }_{x}\rangle \langle {\Psi }_{x}| {\hat{\Delta }}_{i}| {\Psi }_{i}\rangle }{{E}_{x}-{E}_{i}-{\omega }_{i}-i\Gamma },$$

where the $\left\vert {\Psi }_{x}\right\rangle$ denote all intermediate states with a single core hole (energy *E*_*x*_), while ${\hat{\Delta }}_{i}$ (${\hat{\Delta }}_{f}$) represents the light–matter interaction involving the incoming (outgoing) photon. We go beyond the dipole approximation by incorporating the momentum of the photons and representing the dipole operator by the momentum operator $\hat{{{{\bf{p}}}}}$. The short lifetime *τ*_ch_ of the core hole enters Eq. (2) as the broadening parameter Γ = 1/(2*τ*_ch_).

Evaluating the RIXS cross-section (1) from Eq. (2) is an intricate problem, as the interaction of the electrons both in the core levels and in the valence bands plays a role. Furthermore, the creation of a strongly localized core hole is accompanied by strong electrostatic interactions, which give rise to strongly bound excitons and can trigger collective excitations such as phonons and plasmons. In this work, the light–matter interaction is the key ingredient, while details of the core electrons only play a minor role. Therefore, we treat the band electrons on the level of density-functional theory (DFT), which captures the detailed electronic structure, while tabulated values for the deep core levels are used^46,47^. In practice, DFT can capture the multiplet structure of the intermediate and final states only approximately. While the final states correspond to particle-hole excitations, which can be described well within DFT, the relaxation effects in the presence of the core holes render the intermediate states more correlated. The combination with a linear-response treatment of relaxation effects has been shown to yield reasonable agreement with experiments^48,49^ for molecules. For weakly and moderately correlated systems such as perovskites^50^, solving the Bethe-Salpeter equation (BSE) to describe the intermediate states has been successful^51^. Here we use a simplified approach in a similar spirit, where core-hole excitons are included in our theory by considering the core-hole Coulomb interaction *U*^*c*^. Employing the mean-field approximation allows to explicitly solve for the intermediates states $\left\vert {\Psi }_{x}\right\rangle$ using the exciton formalism^52^, and these states are then used to evaluate the RIXS amplitude (2).

By using the mean-field approximation the position of the absorption edge (determined by the exciton binding energy) is only described qualitatively. However, the essence of core-hole relaxation effects and their impact on the CD-RIXS signal are included in our theory. We have ascertained the robustness of our results by varying *U*^*c*^. Together with the first-principle treatment of the light–matter interaction, our key findings are expected to hold when employing more refined simulations.

### Orbital excitations with circular photons

Before presenting the simulated RIXS spectra for specific materials, we discuss how circularly polarized photons can reveal local magnetic orbital excitations. To this end, we analyze the core ingredient of the RIXS amplitude (2): the transition matrix elements. To induce transitions to magnetic *d* orbitals, the core states need to have *p* orbital character (at least for the dominant dipole transitions). The spin–orbit coupling (SOC) in the core splits the core levels in the 2*p* shell into two distinct groups, 2*p*_5/2_ (coined L_2_ edge) and 2*p*_3/2_ (L_3_ edge). We focus on the L_2_ edge here. Exciting from the 3*p* shell (which splits into the M_2_ and M_3_ edge, respectively) is similar in principle, albeit enhanced correlation effects complicate the picture.

Assuming a fully local picture, we consider the transition (see Fig. 1b) from the ground state $\left\vert {\Psi }_{i}\right\rangle$ (no core holes, a single occupied *d* orbital with magnetic quantum number *m*_*f*_) to intermediate states $\left\vert {\Psi }_{x}\right\rangle$ (one core hole, additional electron in the *d* shell with quantum number *m*_*i*_) to final states $\left\vert {\Psi }_{f}\right\rangle$ (*d* electron with quantum number *m*_*f*_ filling the core hole). For illustrative purposes, we use the ultra-short core-hole lifetime (UCL) approximation Γ → *∞* here. We note, however, that the simulated RIXS spectra below are obtained from the full expression for the RIXS amplitude (2). Within the UCL, the RIXS probability simplifies to

3 $$P({m}_{i},{m}_{f})\propto \frac{1}{{\Gamma }^{2}}{\left\vert \mathop{\sum}\limits_{x}\langle {\Psi }_{f}| {\hat{\Delta }}_{f}^{{\dagger} }| {\Psi }_{x}\rangle \langle {\Psi }_{x}| {\hat{\Delta }}_{i}| {\Psi }_{i}\rangle \right\vert }^{2}.$$

The transition probability (3) depends on the polarization of the incoming (outgoing) light **e**_*i*_ (**e**_*f*_). As in experiments, we average over the polarization of the scattered photons. We can now understand which transitions *m*_*i*_ → *m*_*f*_ are driven by circularly polarized incoming photons. Figure 1c—obtained within the independent-electron approximation in the geometry as described below—shows the difference *P*_CD_(*m*_*i*_, *m*_*f*_) = *P*_LCP_(*m*_*i*_, *m*_*f*_) − *P*_RCP_(*m*_*i*_, *m*_*f*_) of the RIXS probability (3) with respect to left-hand circular polarization (LCP) and right-hand circular polarization (RCP). We observe the following general trends: (i) Except for the *m*_*i*_ = 0 case, the circular dichroism is always positive (negative) for *m*_*i*_ > 0 (*m*_*i*_ < 0). Hence, the dichroic signal *P*_CD_(*m*_*i*_, *m*_*f*_) is a direct map of the local orbital angular momentum (OAM) of the *unoccupied* states *m*_*i*_. (ii) For *m*_*i*_ = 0, one finds positive (negative) dichroism for *m*_*f*_ = −2 (*m*_*f*_ = +2). In this case, the dichroic signal is sensitive to the magnetic state of the *occupied* orbitals *m*_*f*_. While the dichroic selection rules illustrated in Fig. 1c have been obtained for a noninteracting electron model, they are expected to also determine the chiral optical properties of the intermediate excitonic states, as has been shown for excitons in two-dimensional semiconductors^53,54^.

### Circular RIXS from monolayer MoSe_2_

**Fig. 2:**
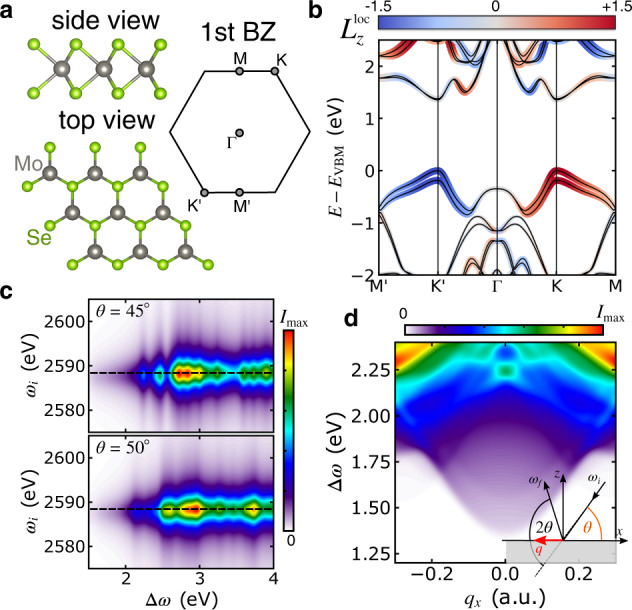
Properties and RIXS spectrum of monolayer MoSe_2_. **a** Sketch of the lattice structure of MoSe_2_ and the first Brillouin zone, including the high-symmetry points. **b** Band structure relative to the valence band maximum (VBM) along the indicated path. The colored fat bands represent the weight of the Mo-*d* orbitals (size) and the local angular momentum (color map). **c** RIXS map showing the *d*-*d* particle-hole excitations at incidence angle *θ* = 45^∘^ (top) and *θ* = 50^∘^ (bottom panel). **d** Dispersion of the particle-hole excitations at fixed incoming photon energy as indicated by the horizontal dashed line in **c**. The inset on the bottom right illustrates the geometry of the calculation: the angle 2*θ* = 130^∘^ is fixed, while varying *θ* corresponds to scanning over the in-plane momentum transfer *q*. The photon energy *ω*_*i*_ is tuned to the L_2_ edge.

With this intuition, we can now investigate RIXS with circular photons from relevant *d*-electron materials. First, we consider monolayer MoSe_2_, a two-dimensional TMDC with remarkable spin polarization, Berry curvature, and OAM^55^. The lattice structure and the first Brillouin zone (BZ) are sketched in Fig. 2a. The band structure (see Fig. 2b) exhibits two top valence bands split by SOC with almost pure, opposite spin polarization along *z* at K and K^′^, respectively. The electronic properties around the K and K^′^ valleys—the most important region due to the direct band gap—is dominated by the Mo-*d* orbitals, as shown by the width of the colored lines in Fig. 2b. The orbital character of the top valence band around the K (K^′^) point is dominated by the ${d}_{+2}=({d}_{{x}^{2}-{y}^{2}}+i{d}_{xy})/\sqrt{2}$ (${d}_{-2}=({d}_{{x}^{2}-{y}^{2}}-i{d}_{xy})/\sqrt{2}$) orbital with an admixture of ${d}_{0}={d}_{{z}^{2}}$ further away from the Dirac points. Hence, the OAM associated with the Mo atoms, ${L}_{z}^{{{{\rm{loc}}}}}$, is very pronounced (color coding in Fig. 2b). The bottom conduction bands are dominated by the ${d}_{{z}^{2}}$ orbital at K (K^′^) with a growing contribution from *d*_+2_ (*d*_−2_) away from the valley center. The local OAM of the top valence band is the dominant contribution to the total OAM, which in turn determines the Berry curvature of the valence bands. Due to time-reversal symmetry, the Berry curvature and the OAM at K (K^′^) has the opposite sign at K^′^ (K).

We chose MoSe_2_ as a representative of the TMDCs of type MX_2_ because RIXS from the Mo 2*p* shell is straightforward to measure, as demonstrated in ref. ^56^ for various compounds. Furthermore, the photon energy in the tender X-ray regime allows for incidence angles close to normal incidence, which reduces geometric effects and enables an interpretation of the CD signal in terms of the magnetic quantum numbers *m*_*i*_, *m*_*f*_. Hence, with X-ray photons tuned to the L_2_ (or L_3_) edge of Mo, the RIXS signal from MoSe_2_ is expected to qualitatively follow the scenario described by Fig. 1c. To confirm this picture, we have calculated the RIXS spectra (L_2_ edge) from Eq. (1) and Eq. (2) with first-principle input. Details are given in the Materials and Methods section. The only parameters are the local Coulomb interaction *U*^*c*^ between the core hole and band electrons localized at the Mo sites and the inverse lifetime Γ. We fix *U*^*c*^ = 8 eV and Γ = 3 eV, which can be estimated from the effective charge *Z* of the core states by assuming a quadratic scaling with *Z*^−2^. Figure 2c shows typical RIXS maps (polarization-averaged) for two different incidence angles *θ*. The resonant behavior with respect to *ω*_*i*_ is due to the resonant structure in the RIXS amplitude (2). Inspecting the dependence on the energy loss Δ*ω* we notice a clear difference between the spectra for different *θ*, which indicates a dispersion of the underlying excitations. To extract this dispersion of the particle-hole excitations in the Mo-*d* orbitals, we focus on the resonant region (black dashed line in Fig. 2c) to maximize the intensity in analogy to experiments. The dispersion of the orbital particle-hole excitations is computed in analogy to typical experimental setups^27^ (illustrated in the inset in Fig. 2d). The in-plane momentum transfer *q* is determined by the incidence angle *θ*, while the scattering angle 2*θ* remains fixed. Varying *θ* and converting to *q* then yields the dispersion presented in Fig. 2d. In the absence of strong correlations in the valence bands, the dispersive RIXS signal in Fig. 2d can be understood as originating from transitions from the occupied valence bands to the conduction bands with momentum change *q*_*x*_. In reality, exciton features would appear at Δ*ω* below the band gap, which needs to be separated out from the signal to focus on the particle-hole excitations, as discussed in ref. ^57^. For *q*_*x*_ ≈ 0, the computed signal is dominated by almost vertical transitions across the direct band gap, while the combined dispersion of the top valence and bottom conduction band determines the dispersion of the orbital excitations.

**Fig. 3:**
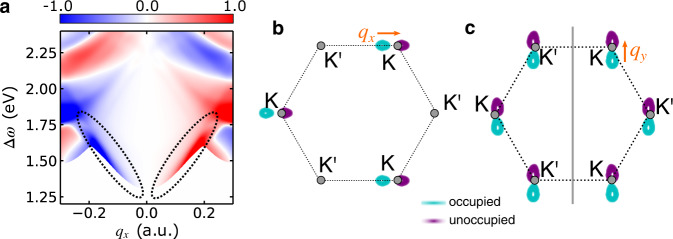
Circular dichroism in RIXS from MoSe_2_. **a** Normalized circular dichroism of the RIXS cross-section (*I*_LCP_ − *I*_RCP_)/(*I*_LCP_ + *I*_RCP_) of monolayer MoSe_2_. *I*_LCP_ (*I*_RCP_) denotes the RIXS intensity for left-circular (right-circular) polarization. The incoming photon energy *ω*_*i*_ and the geometry is the same as in Fig. 2d. The ellipses indicate the region where the OAM of the top valence band dominates the signal. **b** Sketch of the phase space of the *d*-*d* excitations permitted by energy and momentum conservation at fixed momentum transfer *q*_*x*_ = 0.164 a.u. and energy loss Δ*ω* = 1.73 eV. The scattering plane is spanned by the *x* and the *z* direction. **c** Analogous sketch for momentum transfer *q*_*y*_ along the *y* direction. The vertical gray line indicates the mirror plane.

At small momentum transfer, the excitations are predominantly ${d}_{\pm 2}\to {d}_{{z}^{2}}$. From Fig. 1c, we expect circular dichroism in the RIXS process for these orbital transitions, which—due to the almost one-to-one correspondence between local OAM and Berry curvature—reflects the Berry curvature of the top valence band. However, the total Berry curvature around K and K^′^ is opposite, so these contributions cancel out when integrating over the BZ. In such a situation, can there still be circular dichroism in the RIXS process? The answer is affirmative, as demonstrated by Fig. 3a.

While the dichroic signal vanishes at *q*_*x*_ = 0, there is pronounced dichroism for *q*_*x*_ ≠ 0 that changes sign with *q*_*x*_. This can be understood by inspecting the momentum and energy conservation of the RIXS process. Starting from a valence state with energy *ε*_**k***v*_, excitations are only allowed to conduction states with energy *ε*_**k**+**q***c*_ = *ε*_**k***v*_ + Δ*ω*. The breaking of inversion symmetry gives rise to anisotropic electronic orbital textures with respect to **q**. This anisotropy leads to an imbalance between the contributions from the inequivalent K and K^′^ valleys, as illustrated in Fig. 3b. In the case of the dichroic features highlighted by the dashed ellipses in Fig. 3a, the RIXS signal is predominantly determined by transitions at the K^′^ (K) points for *q*_*x*_ > 0 (*q*_*x*_ < 0), where the valence band exhibits *d*_−2_ (*d*_+2_) orbital character. Consistent with Fig. 1c, positive circular dichroism is observed for *q*_*x*_ > 0, and the result reverses upon reversing the sign of *q*_*x*_.

For larger ∣*q*_*x*_∣ other bands start contributing to the RIXS signal, and the simple picture of ${d}_{\pm 2}\to {d}_{{z}^{2}}$ transitions breaks down. Instead, the circular dichroism is governed by the magnetic quantum number *m*_*i*_ corresponding to the OAM of the conduction band, while the valence band character has almost no influence, consistent with scenario (ii) outlined in the discussion of Fig. 1.

Changing the scattering plane to the *y*–*z* plane, on the other hand, leads to exactly vanishing circular dichroism. As illustrated in Fig. 3c, the contribution from the K and K^′^ valleys is identical in this case. The mirror symmetry of the lattice structure with respect to the *y*-axis (Fig. 2a) manifests as a mirror symmetry in reciprocal space, as indicated by the gray vertical line in Fig. 3c. As the mirror operation swaps K ↔ K^′^, there can be no difference in the contribution from K and K^′^.

Hence, by chosing the momentum transfer along the *x* direction, one can achieve valley selectivity and extract the valley-resolved Berry curvature. Controlling the phase space of the particle-hole excitations by varying **q** is a universal principle that applies to systems with broken inversion symmetry.

### Tracing the tunable Berry curvature dipole in 1T^′^-MoS_2_

With the RIXS signal from the prototypical TMDC MoSe_2_ qualitatively understood, we now investigate monolayer 1T^′^-MoS_2_, which is a quantum-spin Hall effect insulator (QSHI)^58^. The most studied representative of this class of TMDCs is 1T^′^-WTe_2_^42,42,44,58^, which is currently considered one of the most robust monolayer QSHI systems^45,59^. However, its band gap is very sensitive to strain. 1T^′^-MoS_2_, on the other hand, has a larger band gap, but is slightly less mechanically stable, which is why most experiments on the 1T^′^ TMDCs have been performed on WTe_2_. Since measuring RIXS from the W 2*p* core-shell is challenging, we present results for the simulated RIXS spectra for 1T^′^-MoS_2_ here. The synthesis of monolayer 1T^′^-MoS_2_ has already been achieved^60^, so measuring the RIXS signal from this material should be feasible. For completeness, we also present results for 1T^′^-WTe_2_ in Supplementary Figs. 1, 2.

**Fig. 4:**
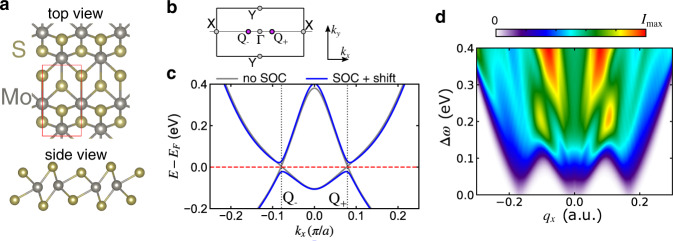
Electronic structure and RIXS spectrum of 1T^′^-MoS_2_. **a** Sketch of the lattice structure. The red rectangle indicates the unit cell. **b** First BZ with important momenta. **c** Band structure with and without including SOC. Without SOC, there is a pair of Dirac cones at Q_±_. **d** Simulated polarization-averaged RIXS intensity at temperature *T* = 20 K. The scattering angle is fixed at 2*θ* = 130^∘^.

The crystal structure (Fig. 4a) possesses inversion symmetry, which excludes any momentum-resolved OAM. The band structure along the *k*_*x*_ direction (Fig. 4b) without SOC features a pair of Dirac cones located at the *Q*_±_ points that gap out upon switching on SOC (see Fig. 4c). In contrast to other 1T^′^ TMDCs, the electronic structure, including a good estimate of the band gap, can be obtained from DFT without the need for corrections^58^. We computed the RIXS signal at the L_2_ and L_3_ edges in an analogous fashion as described for MoSe_2_. The calculated spectrum (Fig. 4d) for momentum transfer along the *x* direction is a direct manifestation of the specifics of the band structure: for small energy loss Δ*ω* < 0.1 eV, there are vertical transitions (*q*_*x*_ = 0) and transitions at *q*_*x*_ = ± 0.18 a.u., which correspond to excitations of the electrons around *Q*_−_ (*Q*_+_) to *Q*_+_ (*Q*_−_). At a low enough temperature (we set *T* = 20 K) and with the energy resolution used in the simulations (~50 meV), transitions below Δ*ω* ≈ 50 meV are suppressed.

**Fig. 5:**
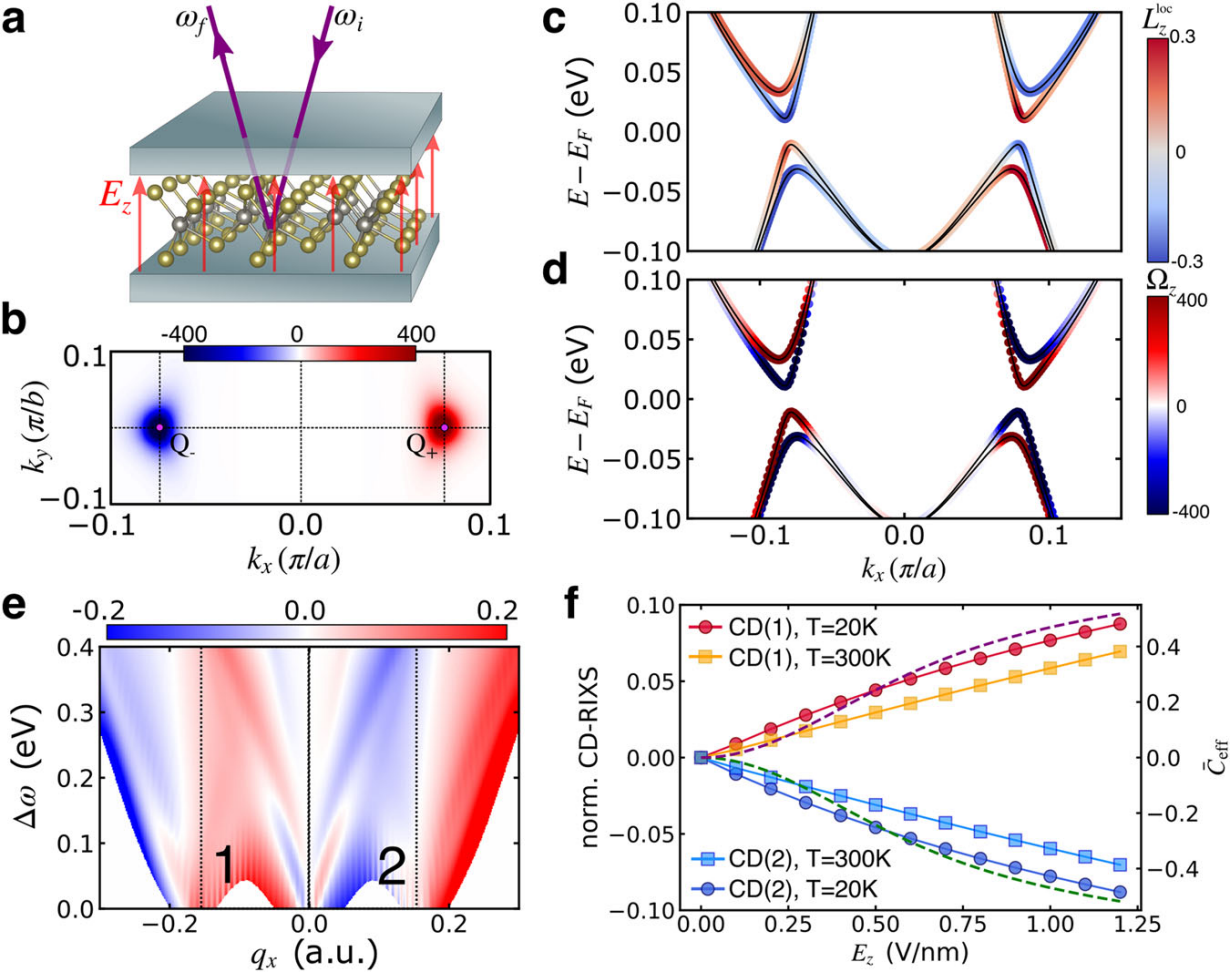
Field-induced Berry curvature and circular dichroism from 1T^′^-MoS_2_. **a** Sketch of the setup for observing electrically switchable Berry curvature by RIXS. **b** Berry curvature Ω_*z*_ of the bottom conduction bands in the vicinity of the *Q*_±_ points for *E*_*z*_ = 0.4 V/m. **c** Fat-band representation of the local OAM ${L}_{z}^{{{{\rm{loc}}}}}$ (color map) along the X-Γ-X path for *E*_*z*_ = 0.4 V/m. **d** Similar to **c**, but the color indicates the Berry curvature Ω_*z*_. **e** Normalized CD-RIXS signal (*I*_LCP_ − *I*_RCP_)/(*I*_LCP_ + *I*_RCP_) for 2*θ* = 130^∘^ and unpolarized outgoing photons. The rectangles denoted by 1 and 2, respectively, represent the phase space where almost-direct transitions to the bottom conduction band (CB) are the dominant processes. **f** Integrated CD-RIXS signal (normalized over the total intensity) in the regions of interest 1, 2 in **e** (symbols), compared to the Berry curvature ${\bar{C}}_{{{{\rm{eff}}}}}$ of the bottom CB integrated region of the BZ shown in **b**.

One of the most remarkable properties of 1T^′^-MoS_2_ is the tunable Berry curvature upon applying an out-of-plane electric field (see Fig. 5a), which can be realized in a hetereostructure^6^. With a sizable electric field *E*_*z*_, the inversion symmetry is broken, which includes an imbalance of the otherwise equivalent Mo atoms. As a result, the spin degeneracy is lifted. The resulting spin texture is locked to the OAM texture, which is the source of the emerging Berry curvature (Fig. 5b). Time-reversal symmetry dictates the total Berry curvature to vanish. The system acquires a Berry curvature dipole which results in a nonlinear Hall response^61^. As shown in Fig. 5b, the bottom conduction band (CB) shows strong Berry curvature (which is opposite for now spin-split bands), which is almost proportional to the OAM of the projections of the Bloch states onto the Mo sites (see Fig. 5c, d). The magnitude of the Berry curvature and the Berry curvature dipole grows upon increasing the field strength up to the point where the CB and valence bands (VB) touch. At this point, the system undergoes a topological phase transition accompanied by a gap closing at the critical field strength of ${E}_{z}^{{{{\rm{crit}}}}}\approx 0.9$ V/nm. The value ${E}_{z}^{{{{\rm{crit}}}}}=0.9$ V/nm is smaller than the one obtained in ref. ^58^. We include the external electric field directly without taking any additional screening into account, which slightly overemphasizes the effects of the electric field. While the spin-Chern number drops to zero^58^, the (charge) Berry curvature continues to grow upon increasing *E*_*z*_.

Inspecting the CD signal for momentum transfer **q** along the *x*-axis (Fig. 5e) we notice pronounced dichroism in the entire *q*_*x*_-Δ*ω* phase space stemming from now strongly asymmetric spectral lobes. In particular, there is a strong CD for small ∣*q*_*x*_∣ at the fringes of the lobes, which is associated with transitions to the kinks of the CB with maximal Berry curvature (highlighted by the rectangles in Fig. 5e). Consistent with the scenario (ii) outlined above, the CD signal predominantly originates from the magnetic states of the unoccupied states, while the magnetic quantum number of the occupied orbitals is less relevant.

Next, we compare the CD signal in regions of interest directly to the Berry curvature. Since the two CBs are almost degenerate away from *Q*_±_, rendering their opposite Berry curvature indistinguishable, we computed the effective Berry curvature by averaging over an energy window:

4 $${\bar{C}}_{{{{\rm{eff}}}}}^{\pm }=\mathop{\sum}\limits_{\alpha }\int\,d{{{\bf{k}}}}\,{\Omega }_{z,\alpha }({{{\bf{k}}}}){w}_{\alpha }({{{\bf{k}}}}),$$

where the integration range is the part of the BZ shown in Fig. 5b. The sum over *α* includes the two CBs, and *w*_*α*_(**k**) is a Gaussian weight factor around the bottom CB. Definition (4) is analogous to the valley Chern number^62^; the averaging procedure reduces ${\bar{C}}_{{{{\rm{eff}}}}}^{\pm }$ for small *E*_*z*_ and thus almost degenerate CBs, which reflects the suppression of observable experimental effects for *E*_*z*_ → 0^6^. Switching on the electric field, the effectively integrated Berry curvature (4) grows approximately linearly in magnitude (Fig. 5f); ${\bar{C}}_{{{{\rm{eff}}}}}^{+}$ (${\bar{C}}_{{{{\rm{eff}}}}}^{-}$) is positive (negative).

Strikingly, the CD-RIXS signal shows the same trend. For a direct comparison, we integrated the CD signal in boxes 1, 2 in Fig. 5f and normalized it by the corresponding polarization-averaged intensity. At low temperatures (circles in Fig. 5f), the CD signal is very close to the integrated Berry curvature. The non-monotonic behavior of the total Berry curvature is also captured. Increasing the temperature to *T* = 300 K—which is on the order of the band gap—the normalized CD-RIXS signal is only slightly reduced.

Similar to MoSe_2_, the finite momentum transfer selects the phase space of the particle-hole excitations. For small *q*_*x*_ > 0 (*q*_*x*_ < 0), transitions from the VB to the CB are only possible close to *Q*_+_ (*Q*_−_). For this reason, excitations occur only in regions with positive (negative) Berry curvature, thus providing momentum-resolved topological properties.

## Discussion

We have presented calculations of CD-RIXS from molybdenum-based TMCDs, in particular monolayer MoSe_2_ and the QSHI 1T^′^-MoS_2_. CD-RIXS is sensitive to the OAM of the orbitals involved in the particle-hole excitations in the Mo-*d* manifold; the magnetic character of the conduction band plays the dominant role. Unlike simple magnetic materials, the relevant bands possess an OAM texture, i.e., a momentum dependence of the relative contributions of the magnetic *d* orbitals. The OAM texture is a signature of Berry curvature, which renders CD-RIXS a powerful tool to investigate the quantum geometric and topological properties of materials. Even for nonmagnetic materials with vanishing total Berry curvature, the CD signal can be pronounced at finite momentum transfer, which gives CD-RIXS an advantage over optical spectroscopies such as Raman spectroscopy. Furthermore, the orbital character of the unoccupied bands is the predominant factor determining the CD. Hence, CD-RIXS yields insights into the OAM texture of the conduction bands. This is a clear advantage over ARPES, as access to the conduction bands (and their orbital properties) is only possible within pump-probe photoemission^63,64^. Moreover, extracting the OAM from CD-RIXS is straightforward due to the selection rules. In contrast, the manifestation of Berry curvature in CD-ARPES is much more involved, as extrinsic effects such as the final-state effects or the experimental geometry complicate the interpretation. The insensitivity of the photons to external electric fields allows to study field-induced transitions, as demonstrated by the switchable Berry curvature in 1T^′^-MoS_2_. The site specificity of RIXS also provides information on the localization of the Bloch wave function^65^, which is directly connected to the band topology^66^.

The idea of selecting the phase space of particle-hole excitations by controlling the momentum transfer **q** is general and can be applied to many other materials. The only requirement is an anisotropic band structure with respect to the direction of **q**. This is generically the case in systems with broken inversion symmetry (which is required for nonvanishing Berry curvature if time-reversal symmetry is present). For the Berry curvature to be reflected in the local OAM, localized orbitals are required, as is typically the case for bands with *d* or *f* orbital character. Investigating topological properties with CD-RIXS is thus expected to be applicable to a large class of materials.

RIXS typically probes a number of many-body excitations besides the particle-hole transitions from the valence to the conduction bands. The main challenge for extracting the information on the OAM texture will be the separation of the dispersive particle-hole continuum from other excitations. Local excitations are typically reflected in a strong RIXS signal below the band gap; their contribution can be isolated from the particle-hole *d*–*d* transitions by careful analysis^57^. For TMDCs in particular, excitons are pronounced but delocalized in space^67^, which should reduce their spectral weight in RIXS spectra. Furthermore, inelastic scattering and relaxation processes in the valence band result in a delocalized response of the electronic structure termed fluorescence, which can be dominant, especially in metallic systems. Removing the fluorescence background is challenging but possible^68^; for molybdenum-based compounds, it has been demonstrated that the main spin–orbit-split peaks are visible on top of the fluorescent line^56^, which supports the feasibility of the proposed CD-RIXS experiment. Apart from magnetic particle-hole excitations, studying the chirality of many-body excitations such as excitons^54^ with CD-RIXS is an interesting perspective.

The recent development of time-resolved RIXS^69–71^ underlines the potential for tracing out-of-equilibrium phenomena. In parallel, several realistic theoretical proposals for using time-resolved RIXS to study light-driven materials by RXIS^72–74^ have been put forward. Combining our CD-RIXS analysis with time-resolved RIXS is thus expected to open a new route for exploring light-induced topological phase transitions^75,76^.

## Methods

### Calculation of the RIXS cross-section

We compute the RIXS intensity from the Kramers–Heisenberg formula in the language of many-body states:

5 $$I({\omega }_{i},{{{{\bf{q}}}}}_{i},{\omega }_{f},{{{{\bf{q}}}}}_{f})=\mathop{\sum}\limits_{f}{\left\vert {A}_{fi}({\omega }_{i},{{{{\bf{q}}}}}_{i},{{{{\bf{q}}}}}_{f})\right\vert }^{2}\delta ({E}_{i}+\Delta \omega -{E}_{f}).$$

Here, *E*_*i*_ (*E*_*f*_) denotes the energy of the ground (final) state, while Δ*ω* = *ω*_*i*_ − *ω*_*f*_ is the energy transfer. The RIXS amplitude *A*_*fi*_(*ω*_*i*_, **q**_*i*_, **q**_*f*_) is defined by

6 $${A}_{fi}({\omega }_{i},{{{{\bf{q}}}}}_{i},{{{{\bf{q}}}}}_{f})=\mathop{\sum}\limits_{{{{\bf{R}}}}}{e}^{-i{{{\bf{q}}}}\cdot {{{\bf{R}}}}}\mathop{\sum}\limits_{x}\frac{\langle {\Psi }_{f}| {\hat{\Delta }}_{f,{{{\bf{R}}}}}^{{\dagger} }| {\Psi }_{x}\rangle \langle {\Psi }_{x}| {\hat{\Delta }}_{i,{{{\bf{R}}}}}| {\Psi }_{i}\rangle }{{E}_{x}-{E}_{i}-{\omega }_{i}-i\Gamma },$$

where *x* labels all intermediate excited states $\left\vert {\Psi }_{x}\right\rangle$ with energy *E*_*x*_; the light–matter interaction with respect to the incoming (outgoing) photon at lattice site **r** is described by ${\hat{\Delta }}_{i,{{{\bf{R}}}}}$ (${\hat{\Delta }}_{f,{{{\bf{R}}}}}$). The momentum transfered to the material is denoted by **q** = **q**_*i*_ − **q**_*f*_.

The many-body states in Eq. (6) are calculated from a Hamiltonian composed of band electrons (${\hat{H}}_{b}$), core electrons (${\hat{H}}_{c}$), and their interaction (${\hat{H}}_{{{{\rm{int}}}}}$):

7 $$\hat{H}={\hat{H}}_{b}+{\hat{H}}_{c}+{\hat{H}}_{{{{\rm{int}}}}}.$$

The Hamiltonian ${\hat{H}}_{b}$ describing the band electrons is constructed in the relevant orbital space from density-functional theory (DFT) as detailed below. The core electrons are described by

8 $$\begin{array}{rcl}{\hat{H}}_{c}&=&{\lambda }_{{{{\rm{SOC}}}}}\mathop{\sum}\limits_{{{{\bf{R}}}}}\mathop{\sum}\limits_{m{m}^{{\prime} }}\mathop{\sum}\limits_{\sigma {\sigma }^{{\prime} }}\langle {\ell }_{c}m\sigma | \hat{{{{\bf{L}}}}}\cdot \hat{{{{\bf{S}}}}}| {\ell }_{c}{m}^{{\prime} }{\sigma }^{{\prime} }\rangle {d}_{{{{\bf{R}}}}m\sigma }^{{\dagger} }{d}_{{{{\bf{R}}}}{m}^{{\prime} }{\sigma }^{{\prime} }}\\ &&+{E}_{c}\mathop{\sum}\limits_{{{{\bf{R}}}}}\mathop{\sum}\limits_{m\sigma }{\hat{n}}_{{{{\bf{R}}}}m\sigma }^{c},\end{array}$$

where ${d}_{{{{\bf{R}}}}m\sigma }^{{\dagger} }$ (*d*_**R***mσ*_) is the creation (annihilation) operator of a core electron in a state with magnetic quantum number *m* and spin *σ*. The energy levels are solely determined by the spin–orbit coupling *λ*_SOC_, the angular momentum quantum number *ℓ*_*c*_, and the energy shift *E*_*c*_. We adjust *λ*_SOC_ and *E*_*c*_ such that the core levels reproduce tabulated values for the edge energies^46,47^.

The interaction of the core and valence electrons is parameterized by

9 $${\hat{H}}_{{{{\rm{int}}}}}=-{U}^{c}\mathop{\sum}\limits_{{{{\bf{R}}}}}\mathop{\sum}\limits_{j}\mathop{\sum}\limits_{m\sigma }{n}_{{{{\bf{R}}}}j}^{b}{d}_{{{{\bf{R}}}}m\sigma }{d}_{{{{\bf{R}}}}m\sigma }^{{\dagger} },$$

where the sum over valence orbitals *j* is restricted to the atoms where the core hole is created; ${n}_{{{{\bf{R}}}}j}^{b}$ denotes the density operator of the band electrons. We treat the interaction (9) on the Hartree–Fock (HF) level, which incorporates the basic physics of bound core-valence excitons^52,77,78^. Consistent with the HF approximation, the initial and the final states are computed without the interaction term: $({\hat{H}}_{b}+{\hat{H}}_{c})\left\vert {\Psi }_{i,f}\right\rangle ={E}_{i,f}\left\vert {\Psi }_{i,f}\right\rangle$. Hence $\left\vert {\Psi }_{i,f}\right\rangle$ is constructed as a determinant of the occupied Kohn–Sham states. The intermediates states can be constructed as a superposition of single-particle excitation from the initial state:

10 $$\left\vert {\Psi }_{x}\right\rangle =\mathop{\sum}\limits_{\alpha \nu }{A}_{\alpha \nu }^{x}({{{\bf{k}}}}){c}_{{{{\bf{k}}}}\alpha }^{{\dagger} }{d}_{{{{\bf{p}}}}\nu },$$

where ${c}_{{{{\bf{k}}}}\alpha }^{{\dagger} }$ is the fermionic creation operator with respect to the Bloch state of the valence band (**k***α*), while *d*_*ν*_ is the annihilation operator with respect to the core states (**p***ν*). Inserting the ansatz (10) into the Schrödinger equation $\hat{H}\left\vert {\Psi }_{x}\right\rangle ={E}_{x}\left\vert {\Psi }_{x}\right\rangle$ allows to solve for the exciton amplitudes ${A}_{\alpha \nu }^{x}({{{\bf{k}}}})$. More details are presented in Supplementary Note 1.

### First-principles implementation

We performed DFT calculations with the Quantum Espresso code^79^ at the level of the Perdew–Burke–Ernzerhof (PBE) approximation to the exchange-correlation functional. We used the corresponding full relativistic pseudopotentials from the PseudoDojo project^80^. The ground state calculations were performed on a 12 × 12 Monkhorst-Pack grid of the first Brillouin zone using a plane-wave cutoff of 80 a.u. and a density cutoff of 500 a.u. in a supercell size of 50 a.u. in the out-of-plane direction. We constructed projective Wannier functions (PWFs) using the Wannier90 code^81^, including the Mo-*d* and the chalcogen *p* orbitals on a 15 × 15 Monkhorst-Pack grid.

This procedure yields the Wannier representation used to construct the band Hamiltonian ${\hat{H}}_{b}$. Consistent with the choice of the PWFs, we represent the Wannier orbitals as Slater-type wave functions:

11 $${\phi }_{j}^{b}({{{\bf{r}}}})={R}_{{n}_{j}}({Z}_{j};r){X}_{{\ell }_{j}{m}_{j}}({\Omega }_{{{{\bf{r}}}}}),$$

where ${R}_{{n}_{j}}({Z}_{j};r)$ is a hydrogenic radial function with principal quantum number *n*_*j*_ and effective charge *Z*_*j*_, while *X*_*ℓm*_(Ω_**r**_) denotes the real spherical harmonics.

Similarly, we describe the core states by the atomic orbitals

12 $${\phi }_{m}^{c}({{{\bf{r}}}})={R}_{{n}_{c}}({Z}_{c};r){X}_{{\ell }_{c}m}({\Omega }_{{{{\bf{r}}}}}).$$

The principal quantum number and the effective charge for the molybdenum core electrons are taken from refs. ^46,47^. With orbitals (11) and (12) we compute the optical matrix elements

13 $${M}_{jm}({{{{\bf{e}}}}}_{a},{{{{\bf{q}}}}}_{a})=\int\,d{{{\bf{r}}}}\,{e}^{-i{{{{\bf{q}}}}}_{a}\cdot {{{\bf{r}}}}}{\phi }_{j}^{b}({{{\bf{r}}}}){{{{\bf{e}}}}}_{a}\cdot \hat{{{{\bf{p}}}}}{\phi }_{m}^{c}({{{\bf{r}}}})$$

by expanding the exponential ${e}^{-i{{{{\bf{q}}}}}_{a}\cdot {{{\bf{r}}}}}$ into spherical harmonics, using the Clebsch-Gordan algebra, and calculating the remaining radial integrals. The light–matter coupling operators entering the RIXS amplitude (6) are then expressed as many-body operators by

14 $${\hat{\Delta }}_{a,{{{\bf{R}}}}}=\mathop{\sum}\limits_{j}\mathop{\sum}\limits_{m\sigma }{M}_{jm}({{{{\bf{e}}}}}_{a},{{{{\bf{q}}}}}_{a}){c}_{{{{\bf{R}}}}j\sigma }^{{\dagger} }{d}_{{{{\bf{R}}}}m\sigma },$$

where *a* = *i*, *f* and where ${c}_{{{{\bf{R}}}}j\sigma }^{{\dagger} }$ stands for the creation operator of the band electrons.

The electric field *E*_*z*_ was included by adding the dipole term into the Kohn–Sham Hamiltonian:

15 $${\hat{H}}_{b}({E}_{z})={\hat{H}}_{b}({E}_{z}=0)-q{E}_{z}\mathop{\sum}\limits_{{{{\bf{k}}}}}\mathop{\sum}\limits_{\alpha {\alpha }^{{\prime} }}{D}_{\alpha {\alpha }^{{\prime} }}^{z}({{{\bf{k}}}}){c}_{{{{\bf{k}}}}\alpha }^{{\dagger} }{c}_{{{{\bf{k}}}}\alpha }.$$

Here, ${D}_{\alpha {\alpha }^{{\prime} }}^{z}({{{\bf{k}}}})$ is the dipole matrix element calculated directly from the Wannier functions^82^. Compared to a self-consistent calculation which explicitly includes the electric field, this approach neglects screening effects due to the rearrangement of the density. The impact of the electric field is thus stronger than in reality; compared to the critical field strengths for the topological phase transition from ref. ^58^, we find roughly a factor of 1.5. We stress that upon rescaling the field strength, excellent agreement with the first-principles electronic structure is obtained. Due to the strong Coulomb potential, the electric field can be neglected for the core electrons.

### Supplementary information

Supplemental Material

## Data Availability

The input files for QUANTUM ESPRESSO and WANNIER90, the simulated RIXS spectra, and the scripts to generate the plots in this work are available on the Materials Cloud in the archive 10.24435/materialscloud:ck-7m. The custom computer code used to calculate the RIXS spectra is available upon reasonable request. Band structures, the Berry curvature, and orbital angular momentum were computed using the open-access code dynamics-w90.
