# Supplementary material for: Probing magnetic orbitals and Berry curvature with circular dichroism in resonant inelastic X-ray scattering
Source: NPJ Quantum Mater. 2023 Jan 19;8(1):6. doi: 10.1038/s41535-023-00538-x (PMC11041711; doi:10.1038/s41535-023-00538-x)
Supplement: Supplementary file 1 — Supplemental Material [file 41535_2023_538_MOESM1_ESM.pdf]

# Suplementary Material: Probing magnetic orbitals and Berry curvature with circular dichroism in resonant inelastic X-ray scattering

Michael Schüler,[1,2,3] Thorsten Schmitt,[4] and Philipp Werner[3]
[1]Condensed Matter Theory Group, Paul Scherrer Institute, CH-5232 Villigen PSI, Switzerland
[2]Laboratory for Materials Simulations, Paul Scherrer Institute, CH-5232 Villigen PSI, Switzerland
[3]Department of Physics, University of Fribourg, CH-1700 Fribourg, Switzerland
[4]Photon Science Division, Swiss Light Source, Paul Scherrer Institute, CH-5232 Villigen PSI, Switzerland
(Dated: January 2, 2023)

## SUPPLEMENTARY NOTE 1

In this Supplementary Note we discuss the theory for calculating the spectra presented in the main text in detail.

We consider a Hamiltonian composed of band electrons ($\hat{H}_b$), core electrons ($\hat{H}_c$), and their interaction ($\hat{H}_{\mathrm{int}}$):

$$\hat{H} = \hat{H}_b + \hat{H}_c + \hat{H}_{\mathrm{int}} \ . \tag{1}$$

We assume effectively non-interacting band electrons with orbital index $j$ and spin $\sigma$ described by

$$\hat{H}_b = \sum_{\mathbf{k}} \sum_{jj'} \sum_{\sigma\sigma'} h_{j\sigma j'\sigma'}(\mathbf{k}) c^\dagger_{\mathbf{k}j\sigma} c_{\mathbf{k}j'\sigma'} = \sum_{\mathbf{k}\alpha} \varepsilon^b_{\mathbf{k}\alpha} c^\dagger_{\mathbf{k}\alpha} c_{\mathbf{k}\alpha} \ , \tag{2}$$

while the core electrons are described by

$$\begin{aligned}\hat{H}_c = {} & \lambda_{\mathrm{SOC}} \sum_{\mathbf{R}} \sum_{mm'} \sum_{\sigma\sigma'} \langle \ell_c m\sigma | \hat{\mathbf{L}} \cdot \hat{\mathbf{S}} | \ell_c m'\sigma' \rangle d^\dagger_{\mathbf{R}m\sigma} d_{\mathbf{R}m'\sigma'} \\ & + E_c \sum_{\mathbf{R}} \sum_{m\sigma} \hat{n}^c_{\mathbf{R}m\sigma} \ . \end{aligned} \tag{3}$$

In Eq. (3) we have ignored any dispersion of the core states; their energy levels are solely determined by the spin-orbit coupling $\lambda_{\mathrm{SOC}}$, the angular momentum quantum number $\ell_c$ and the energy shift $E_c$. We adjust $\lambda_{\mathrm{SOC}}$ and $E_c$ such that the core levels reproduce tabulated values for the edge energies.

Band electrons are described by the fermionic operators $c_{\mathbf{k}j\sigma}$, while the core states are described by $d_{\mathbf{R}m\sigma}$; the corresponding number operators are denoted by $n^b_{\mathbf{k}j\sigma}$ and $n^c_{\mathbf{R}m\sigma}$, respectively. Switching to the Wannier representation for the band electrons, the interaction between band and core electrons can be incorporated into the Hamiltonian (1) by

$$\hat{H}_{\mathrm{int}} = -\sum_{\mathbf{R}} \sum_{j} \sum_{m\sigma} U^c_j \hat{n}^b_{\mathbf{R}j} (1 - n^c_{\mathbf{R}m\sigma}) \tag{4}$$

with $\hat{n}_{\mathbf{R}j} = \sum_\sigma \hat{n}_{\mathbf{R}j\sigma}$. The interaction of the core hole and the band electrons $U^c_j \equiv U^c$ is constant if the orbital index $j$ corresponds to the atomic site where the core hole is created and zero otherwise.

While $U^c$ is typically on the order of 10 eV, the large energy difference between the core states and band electrons renders the Hamiltonian (1) weakly correlated, provided that correlations have little effect on the electronic structure near the Fermi level. We invoke this assumption and use density functional theory to construct the band Hamiltonian (2). We assume Slater determinants for the many-body states without a core hole. At this level of description, the intermediate core-band exciton states can be determined exactly and the ground state is simply given by

$$|\Psi_0\rangle = \prod_{\mathbf{k}\alpha\in\mathrm{occ}} c^\dagger_{\mathbf{k}\alpha} \prod_{\mathbf{k}\nu} d^\dagger_{\mathbf{k}\nu} |\mathrm{vac}\rangle \equiv |\Psi^b_0\rangle \otimes |\Psi^c_0\rangle \ . \tag{5}$$

Here, we have introduced the Bloch basis $|\psi_{\mathbf{k}\alpha}\rangle$ as the eigenstates of the single-particle Hamiltonian in Eq. (2) (energies $\varepsilon^b_{\mathbf{k}\alpha}$). Similarly, we define the core states $|\chi_\nu\rangle$ as the eigenstates (with eigenvalue $\varepsilon^c_\nu$) of the single-particle Hamiltonian in Eq. (3) (since there is no dispersion, the eigenstates for different $\mathbf{k}$ are degenerate). The energy of the ground state (5) is given by

$$E_0 = \sum_{\mathbf{k}\alpha\in\mathrm{occ}} \varepsilon^b_{\mathbf{k}\alpha} + \sum_{\mathbf{p}\nu} \varepsilon^c_\nu . \tag{6}$$

In analogy to the ground state (5) we can introduce excited states without any core holes but with band electrons exciteded to the conduction bands:

$$
\begin{aligned}
|\Psi_{\mathbf{k}_1\alpha_1,\mathbf{k}_2\alpha_2}\rangle &= c^\dagger_{\mathbf{k}_1\alpha_1} \prod_{\substack{\mathbf{k}\alpha\in\mathrm{occ}\\ \mathbf{k}\alpha\neq\mathbf{k}_2\alpha_2}} c^\dagger_{\mathbf{k}\alpha} \prod_{\mathbf{k}\nu} d^\dagger_{\mathbf{k}\nu}|\mathrm{vac}\rangle \\
&= c^\dagger_{\mathbf{k}_1\alpha_1} c_{\mathbf{k}_2\alpha_2}|\Psi^b_0\rangle \otimes |\Psi^c_0\rangle \,.
\end{aligned} \tag{7}
$$

The energy of the state (7) is given by

$$
E_{\mathbf{k}_1\alpha_1} = E_0 + \varepsilon^b_{\mathbf{k}_1\alpha_1} - \varepsilon^b_{\mathbf{k}_2\alpha_2} \,. \tag{8}
$$

Finally, we consider states with a single core hole. These states are exciton states, where the unoccupied band states (the core levels) play the role of the conduction (valence) bands in the semiconductor exciton language [1–3]. As shown in Refs. [1, 2, 4], exciton states of the type

$$
|\Psi_{\mathbf{p}\gamma}\rangle = \sum_{\mathbf{k}\alpha\nu} A^{\mathbf{p}\gamma}_{\mathbf{k}\alpha\nu} c^\dagger_{\mathbf{k}\alpha} d_{\mathbf{k}-\mathbf{p}\nu}|\Psi_0\rangle \tag{9}
$$

are exact eigenstates of the Hamiltonian (1) if the coefficients $A^{\mathbf{p}\gamma}_{\mathbf{k}\alpha\nu}$ obey a corresponding eigenvalue equation. The index $\gamma$ labels the exciton bands, while $\mathbf{p}$ represents the momentum of the particle-hole pair. The band indices $\alpha$ are restricted to the unoccupied states. While working with the exciton states (9) is possible, the resulting equation for the exciton amplitudes involve convolutions over the entire Brillouin zone. Due to the degeneracy of the core levels, we can construct a simpler but equivalent set of eigenstates by

$$
|\Psi_{\mathbf{k}\mathbf{p}\lambda}\rangle = \sum_{\alpha\nu} A^\lambda_{\alpha\nu}(\mathbf{k}) c^\dagger_{\mathbf{k}\alpha} d_{\mathbf{p}\nu}|\Psi_0\rangle \,. \tag{10}
$$

It is straightforward to show that superpositions of (10) can be constructed such that the states (9) are recovered.

Applying the Hamiltonian (1) onto the states (10) we obtain a linear combination of the coefficients $A^\lambda_{\alpha\nu}(\mathbf{k})$. Projecting onto states $|\Phi_{\mathbf{k}\mathbf{p}\alpha\nu}\rangle = c^\dagger_{\mathbf{k}\alpha} d_{\mathbf{p}\nu}|\Psi_0\rangle$ this route yields the effective eigenvalue equation

$$
\begin{aligned}
E^\lambda_{\mathbf{k}} A^\lambda_{\alpha\nu}(\mathbf{k}) &= \langle \Phi_{\mathbf{k}\mathbf{p}\alpha\nu}|\hat{H}|\Psi_{\mathbf{k}\mathbf{p}\lambda}\rangle \\
&= (E_0 + \varepsilon^b_{\mathbf{k}\alpha} - \varepsilon^c_\nu - \Delta\varepsilon) A^\lambda_{\alpha\nu}(\mathbf{k}) - \sum_{\alpha'} \mathcal{V}_{\alpha\alpha'}(\mathbf{k}) A^\lambda_{\alpha'\nu}(\mathbf{k}) \,.
\end{aligned} \tag{11}
$$

Here we have introduced the energy shift

$$
\Delta\varepsilon = -\frac{1}{N}\sum_{j\sigma} U^c_j \sum_{\mathbf{k}\alpha\in\mathrm{occ}} |V_{j\sigma\alpha}(\mathbf{k})|^2 \,, \tag{12}
$$

which describes the interaction of the occupied bands with the core hole. The coefficients $V_{j\sigma\alpha}(\mathbf{k})$ are defined by the expansion of the Bloch states $|\psi_{\mathbf{k}\alpha}\rangle$ in terms of Wannier functions:

$$
|\psi_{\mathbf{k}\alpha}\rangle = \frac{1}{\sqrt{N}} \sum_{\mathbf{R}} \sum_{j\sigma} e^{i\mathbf{k}\cdot\mathbf{R}} V_{j\sigma\alpha}(\mathbf{k}) |\phi^b_j(\mathbf{R})\rangle \,. \tag{13}
$$

The coupling term $\mathcal{V}_{\alpha\alpha'}(\mathbf{k})$ is also most conveniently expressed in terms of the Wannier coefficients:

$$
\mathcal{V}_{\alpha\alpha'}(\mathbf{k}) = \frac{1}{N}\sum_{j\sigma} U^c_j V^*_{j\sigma\alpha}(\mathbf{k}) V_{j\sigma\alpha'}(\mathbf{k}) \,. \tag{14}
$$

In summary, upon diagonalizing the effective Hamiltonian $\widetilde{H}_{\alpha\alpha'}(\mathbf{k}) = \varepsilon^b_{\mathbf{k}\alpha}\delta_{\alpha\alpha'} - \mathcal{V}_{\alpha\alpha'}(\mathbf{k})$ (let's denote the corresponding eigenvalues by $\widetilde{\varepsilon}^b_{\mathbf{k}\lambda}$), we obtain the exciton states (10) and the many-body excitation energies

$$
E^\lambda_{\mathbf{k}} - E_0 = \widetilde{\varepsilon}^b_{\mathbf{k}\lambda} - \varepsilon^c_\nu + \Delta\varepsilon \,. \tag{15}
$$

### Exciton theory for the RIXS cross section

To derive a simplified expression for the RIXS intensity $I(\omega_i, \mathbf{q}_i, \omega_f, \mathbf{q}_f)$, we start from the Kramers-Heisenberg formula in the language of many-body states:

$$I(\omega_i, \mathbf{q}_i, \omega_f, \mathbf{q}_f) = \sum_f \left| A_{fi}(\omega_i, \mathbf{q}_i, \mathbf{q}_f) \right|^2 \delta(E_i + \Delta\omega - E_f) \,. \tag{16}$$

Here, $E_i$ ($E_f$) denotes the energy of the ground (final) state, while $\Delta\omega = \omega_i - \omega_f$ is the energy transfer. The RIXS amplitude $A_{fi}(\omega_i, \mathbf{q}_i, \mathbf{q}_f)$ is defined by

$$A_{fi}(\omega_i, \mathbf{q}_i, \mathbf{q}_f) = \sum_x \frac{\langle \Psi_f | \hat{\Delta}^\dagger_f | \Psi_x \rangle \langle \Psi_x | \hat{\Delta}_i | \Psi_i \rangle}{E_x - E_i - \omega_i - i\Gamma} \,, \tag{17}$$

where $x$ labels all intermediate excited states $|\Psi_x\rangle$ with energy $E_x$; the light-matter interaction with respect to the incoming (outgoing) photon is described by $\hat{\Delta}_i$ ($\hat{\Delta}_f$). Due to the strong localization of the involved core states, we assume that the orbital excitations only occur locally on the resonant atom sites. Within this picture, the RIXS amplitude (17) then becomes

$$A_{fi}(\omega_i, \mathbf{q}_i, \mathbf{q}_f) = \sum_{\mathbf{R}} e^{i\mathbf{q}\cdot\mathbf{R}} \sum_x \frac{\langle \Psi_f | \hat{\Delta}^\dagger_{f,\mathbf{R}} | \Psi_x \rangle \langle \Psi_x | \hat{\Delta}_{i,\mathbf{R}} | \Psi_i \rangle}{E_x - E_i - \omega_i - i\Gamma} \,, \tag{18}$$

where $\mathbf{q} = \mathbf{q}_f - \mathbf{q}_i$ is the momentum transfer, while $\Gamma$ denotes the inverse lifetime of the core hole. The light-matter coupling is restricted to the local site $\mathbf{R}$.

### Light-matter coupling

We treat the light-matter coupling within the velocity gauge, which results in a coupling of the vector potential $\mathbf{A}$ to the momentum operator (the diamagnetic term can be neglected). Working in the Coulomb gauge $\nabla \cdot \mathbf{A} = 0$, we can thus treat the light-matter interaction *beyond* the dipole approximation (in contrast to the usual length-gauge approach). To compute optical transitions from localized core levels to the band manifold, we represent the Bloch states in terms of Wannier functions (see Eq. (13)). We also introduce the Wannier representation of the core states:

$$|\chi_{\mathbf{p}\nu}\rangle = \frac{1}{\sqrt{N}} \sum_{\mathbf{R}} \sum_{m\sigma} e^{i\mathbf{k}\cdot\mathbf{R}} C_{m\sigma\nu} |\phi^c_m(\mathbf{R})\rangle \,. \tag{19}$$

In the basis of Wannier orbitals (cf. (13)) and core orbitals (cf. (19)), the light-matter coupling is then defined as

$$\begin{aligned} \hat{\Delta}_{a,\mathbf{R}} &= \sum_{j\sigma} \sum_m \langle \phi^b_j(0) | e^{-i\mathbf{q}_a\cdot\mathbf{r}} \mathbf{e}_a \cdot \hat{\mathbf{p}} | \phi^c_m(0) \rangle c^\dagger_{\mathbf{R}j\sigma} d_{\mathbf{R}m\sigma} \\ &\equiv \sum_{j\sigma} \sum_m M_{jm}(\mathbf{e}_a, \mathbf{q}_a) c^\dagger_{\mathbf{R}j\sigma} d_{\mathbf{R}m\sigma} \,, \end{aligned} \tag{20}$$

where $a = i, f$ and where $\mathbf{e}_a$ denotes the polarization of the photons and $\hat{\mathbf{p}} = -i\nabla$ represents the momentum operator. Using the Wannier representations (13) and (19), the fermionic operators in Eq. (20) can be transformed into the basis of Bloch states and core eigenstates.

We can now calculate the matrix elements in the RIXS amplitude (18). We start with the matrix elements with respect to the incoming photons. We use the ground state (5) as the initial state $|\Psi_i\rangle$. The intermediate states $|\Psi_x\rangle$ have exactly one core hole; we can represent the band-space wave-function by states of the type (10). We label the core hole (excited band electron) state as $(\mathbf{p}_x\nu_x)$ $((\mathbf{k}_x\alpha_x))$. Inserting Eqs. (5) and (10) we find

$$\begin{aligned} \langle \Psi_x | \hat{\Delta}_{i,\mathbf{R}} | \Psi_i \rangle = &\sum_\beta \sum_{j\sigma} \sum_m M_{jm}(\mathbf{e}_i, \mathbf{q}_i) [A^\lambda_{\beta\nu_x}(\mathbf{k}_x)]^* \\ &\times \langle \Psi^b_0 | c_{\mathbf{k}_x\beta} c^\dagger_{\mathbf{R}j\sigma} | \Psi^b_0 \rangle \langle \Psi^c_0 | d^\dagger_{\mathbf{p}_x\nu_x} d_{\mathbf{R}m\sigma} | \Psi^c_0 \rangle \,. \end{aligned}$$

Using the Wannier representations (13),(19) one obtains

$$\langle \Psi^c_0 | d^\dagger_{\mathbf{p}_x\nu_x} d_{\mathbf{R}m\sigma} | \Psi^c_0 \rangle = \frac{1}{\sqrt{N}} e^{i\mathbf{p}_x\cdot\mathbf{R}} C_{m\sigma\nu_x}$$

and

$$\langle\Psi_0^b|c_{\mathbf{k}_x\beta}c^\dagger_{\mathbf{R}j\sigma}|\Psi_0^b\rangle = \frac{1}{\sqrt{N}}e^{-i\mathbf{k}_x\cdot\mathbf{R}}V^*_{j\sigma\beta}(\mathbf{k}) ,$$

since $\beta \in$ unocc. Combining these relations, we can evaluate the matrix element and find

$$\langle\Psi_x|\hat{\Delta}_{i,\mathbf{R}}|\Psi_i\rangle = \frac{1}{N}e^{i(\mathbf{p}_x-\mathbf{k}_x)\cdot\mathbf{R}}\widetilde{M}_{\mathbf{k}_x\lambda,\nu_x}(\mathbf{e}_i,\mathbf{q}_i) , \tag{21}$$

where

$$\widetilde{M}_{\mathbf{k}_x\lambda,\nu_x}(\mathbf{e}_i,\mathbf{q}_i) = \sum_\beta\sum_{j\sigma}\sum_m V^*_{j\sigma\beta}(\mathbf{k}_x)[A^\lambda_{\beta\lambda_x}(\mathbf{k}_x)]^* \times C_{m\sigma\nu_x}M_{jm}(\mathbf{e}_i,\mathbf{q}_i) . \tag{22}$$

The transition matrix element $\langle\Psi_f|\hat{\Delta}^\dagger_f|\Psi_x\rangle$ is obtained in a similar way. The final state $|\Psi_f\rangle$ corresponds directly to Eq. (7). Inserting Eq. (7) and Eq. (10), the matrix element to be computed is

$$\langle\Psi_f|\hat{\Delta}^\dagger_{f,\mathbf{R}}|\Psi_x\rangle = \sum_\beta\sum_{j\sigma}\sum_m \langle\Psi_0^b|c^\dagger_{\mathbf{k}_2\alpha_2}c_{\mathbf{k}_1\alpha_1}c_{\mathbf{R}j\sigma}c^\dagger_{\mathbf{k}_x\beta}|\widetilde{\Psi}_0^b\rangle \times\langle\Psi_0^c|d^\dagger_{\mathbf{R}m\sigma}d_{\mathbf{p}_x\nu_x}|\Psi_0^c\rangle M^*_{jm,\sigma}(\mathbf{e}_f,\mathbf{q}_f)A^\lambda_{\beta\nu_x}(\mathbf{k}_x) .$$

Following the analogous steps as above, we find

$$\langle\Psi_f|\hat{\Delta}^\dagger_{f,\mathbf{R}}|\Psi_x\rangle = \frac{1}{N}\delta_{\mathbf{k}_1,\mathbf{k}_x}e^{-i(\mathbf{p}_x-\mathbf{k}_2)\cdot\mathbf{R}}A^\lambda_{\alpha_1\nu_x}(\mathbf{k}_x) \times M^*_{\mathbf{k}_2\alpha_2\nu_x}(\mathbf{e}_f,\mathbf{q}_f) . \tag{23}$$

Note the overlap term in Eq. (23) that accounts for the relaxation of the band structure upon filling the core hole. The matrix element with respect to the band basis is defined similarly as in Eq. (22).

### RIXS amplitude and cross section

Now we combine the matrix elements (21) and (23) to evaluate the RIXS amplitude (18). The sum over the lattice sites $\mathbf{R}$ yields the momentum conservation

$$\sum_\mathbf{R} e^{i\mathbf{q}\cdot\mathbf{R}}e^{i(\mathbf{p}_x-\mathbf{k}_x)\cdot\mathbf{R}}e^{-i(\mathbf{p}_x-\mathbf{k}_2)\cdot\mathbf{R}} = N\delta_{\mathbf{k}_2+\mathbf{q},\mathbf{k}_x} .$$

Inserting the excitation energy (15) and the expressions for the matrix elements, Eqs. (21) and (23), the RIXS amplitude becomes

$$A_{fi} = \frac{1}{N}\sum_{\mathbf{k}_x\mathbf{p}_x}\sum_{\lambda\nu_x}\frac{M^*_{\mathbf{k}_2\alpha_2\nu_x}(\mathbf{e}_f,\mathbf{q}_f)\widetilde{M}_{\mathbf{k}_2+\mathbf{q}\lambda\nu_x}(\mathbf{e}_i,\mathbf{q}_i)}{\tilde{\varepsilon}^b_{\mathbf{k}_x\lambda}-\varepsilon^c_{\nu_x}+\Delta\varepsilon-\omega_i-i\Gamma} \times A^\lambda_{\alpha_1\nu_x}(\mathbf{k}_x)\delta_{\mathbf{k}_2+\mathbf{q},\mathbf{k}_1}\delta_{\mathbf{k}_1,\mathbf{k}_x} \tag{24}$$

upon summing over all internal degrees of freedom of the exciton states (10).

Finally, we can rename $\mathbf{k}_2\to\mathbf{k}$, $\alpha_2\to\alpha$, $\alpha_1\to\alpha'$, remove the momentum index $\mathbf{k}_1$, and insert Eq. (24) into the RIXS cross section (16):

$$I(\omega_i,\mathbf{q}_i,\omega_f,\mathbf{q}_f) = \sum_\mathbf{k}\sum_{\alpha\alpha'}\left|A_{\alpha\alpha'}(\mathbf{k},\mathbf{q})\right|^2\delta(\varepsilon^b_{\mathbf{k}\alpha}-\varepsilon^b_{\mathbf{k}+\mathbf{q}\alpha'}+\Delta\omega) , \tag{25}$$

where we have relabeled the amplitude (24) by the corresponding internal indices. The sum in Eq. (25) is restricted to $\mathbf{k}\alpha\in$ occ and $\mathbf{k}\alpha'\in$ unocc.

## SUPPLEMENTARY NOTE 2

In analogy to $MoSe_2$ studied in the main text, we have also performed calculations for monolayer $WSe_2$. In contrast to molybdenum, the L edge of tungsten is in the hard X-ray regime, which constrains the angle of incidence in existing experimental setups. We fix $2\theta = 90°$; at photon energies of $\hbar\omega_i \sim 11500$ eV the range of accessible values for the momentum transfer $\mathbf{q}$ is still big enough for dispersive excitations as presented in the main text.

Assuming roughly quadratic scaling of the core-valence interaction $U^c$ and the inverse lifetime $\Gamma$ with the effective charge $Z$, we estimate $U^c = 16$ eV and $\Gamma = 6$ eV. Apart from this difference and the geometry, the calculation is analogous to the case of $MoSe_2$. Supplementary Fig. 1 presents the results for $WSe_2$. The band structure and local OAM texture (Supplementary Fig. 1(a)) is similar to $MoSe_2$, and so is the polarization-averaged RIXS spectrum (Supplementary Fig. 1(b)). The circular dichroism of the RIXS signal (Supplementary Fig. 1(c)) possesses a richer structure than in the case of $MoSe_2$. However, the basic features are analogous: for the dichroic features highlighted by the dashed ellipses in Supplementary Fig. 1(c), the RIXS signal is predominantly determined by transitions at the K′ (K) points for $q_x > 0$ ($q_x < 0$), where the valence band exhibits $d_{-2}$ ($d_{+2}$) orbital character. For larger magnitude of $q_x$, the OAM character of the conduction band dominates the circular dichroism, giving rise to a sign change of the dichroism.

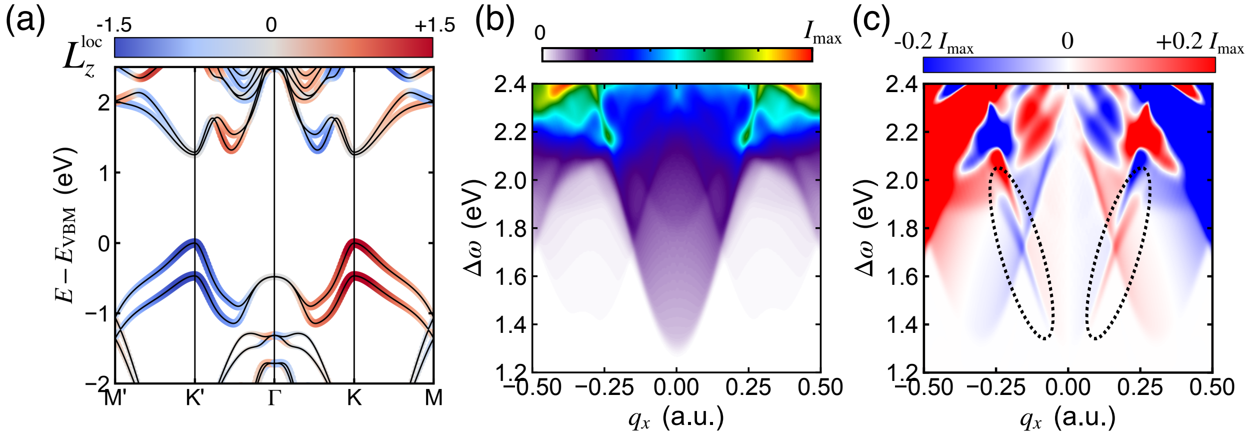


Supplementary Figure 1. **Properties and RIXS spectrum of monolayer $WSe_2$.** (a) Band structure relative to the valence band maximum (VBM) along the indicated path. The colored fat bands represent the weight of the W-$d$ orbitals (size) and the local angular momentum (color map). (c) RIXS map showing the $d$–$d$ excitations. (b) Dispersion of the RIXS spectrum at fixed incoming photon energy $\bar{\omega}_i = 11468$ eV. (c) Circular dichroism of the RIXS cross section. The ellipses indicate the region where the OAM of the top valence band dominates the signal.

## S1. RIXS CALCULATIONS FOR TUNGSTEN DITELLURIDE

We have also investigated monolayer 1T′-$WTe_2$, which is a quantum-spin Hall insulator (QSHI) [5–7]. The crystal structure is analogous to 1T′-$MoS_2$ shown in Fig. 5(a) in the main text. In contrast to 1T′-$MoS_2$, 1T′-$WTe_2$ without SOC features a pair of tilted Dirac cones located at the $Q_\pm$ points that gap out upon switching on SOC (see Supplementary Fig. 2(a)). The PBE functional used in our calculations does not capture the band gap of 1T′-$WTe_2$ correctely, as exchange effects are important [7, 8]. To correct this slight deficiency we include a shift of the conduction bands (scissor operator) in the Wannier Hamiltonian to match the band gap of ≈ 30 meV given in Ref. [9].

The RIXS spectrum is a direct manifestion of the intriguing specifics of the band structure: there are no vertical transitions ($q_x = 0$) for energy loss $\Delta\omega < 0.3$ eV. The main feature of the signal are two lobes that correspond to transitions from the top valence bands with $k_x \approx 0$ to the bottom conduction band at $k_x \approx Q_\pm$.

We also investigated the effects of applying an out-of-plane electric field which was implemented by the saw-like potential in a large supercell; we computed the self-consistent electronic structure and the Wannier functions for each value $E_z = 0.2, 0.4, 0.6, 0.8, 1.0$ V/m. As for 1T′-$MoS_2$, the spin degeneracy is lifted and the Berry curvature in the conduction band becomes pronounced (Supplementary Fig. 2(b)). The Berry curvature corresponds directly to the local OAM with respect to the W sites (Supplementary Fig. 2(c)–(d)). Inspecting the CD signal for momentum transfer $\mathbf{q}$ along the $x$-axis (Supplementary Fig. 2(e)) we notice pronounced dichroism in the entire $q_x$-$\Delta\omega$ phase space stemming from now strongly asymmetric spectral lobes. In particular, there is a strong circular dichroism for small $|q_x|$ at the fringes of the lobes, associated with transitions to the kinks of the CB with maximal Berry curvature (highlighted by the triangles in Supplementary Fig. 2(e)). In Supplementary Fig. 2(f) we compare the circular dichroism integrated over the triangle regions to the effective Berry curvature, determined by

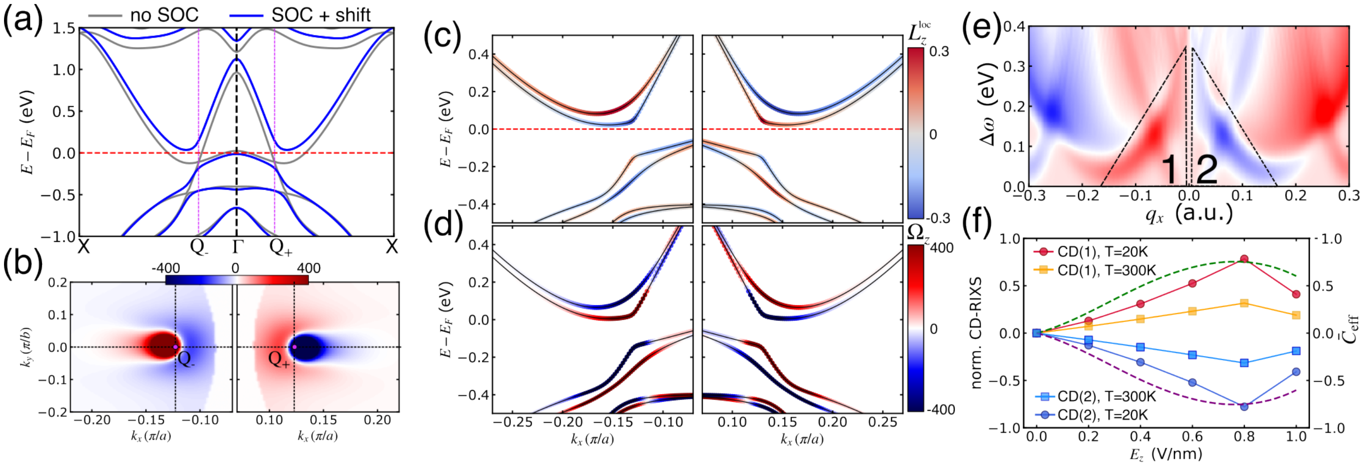

Supplementary Figure 2. **Properties and RIXS spectrum of monolayer 1T′-WTe$_2$.** (a) Band structure with and without including SOC. Without SOC, there is a pair of tilted Dirac cones at Q$_\pm$. (b) Berry curvature $\Omega_z$ of the bottom conduction bands in the vicinity of the $Q_\pm$ points for $E_z = 0.6$ V/m. (c) Fat-band representation of the weight of the W-$d$ orbitals (size of the line) and the local OAM $L_z^{\mathrm{loc}}$ (color map) along the X-Γ-X path for $E_z = 0.6$ V/m. (d) Similar to (c), but the color indicates the Berry curvature $\Omega_z$. (e) CD-RIXS signal for $2\theta = 90°$. The triangles denoted as 1 and 2, respectively, represent the phase space where almost-direct transitions to the bottom conduction band (CB) are the dominant process. (f) Integrated CD-RIXS signal (normalized over the total intensity) in the triangle regions 1, 2 in (e) (symbols), compared to the Berry curvature $\bar{C}_{\mathrm{eff}}$ of the bottom CB integrated over patches around $Q_\pm$ shown in (b).

Eq. (4) in the main text. Switching on the electric field, the effective integrated Berry curvature grows in magnitude with a maximum around $E_z \approx 0.75$ V/m (Supplementary Fig. 2(f)).

The CD-RIXS signal shows the same trend. For a direct comparison we integrated the CD signal in the triangles 1,2 in Supplementary Fig. 2(e) and normalized by the corresponding polarization-averaged intensity. At low temperature (circles in Supplementary Fig. 2(f)), the circular dichroism closely follows the integrated Berry curvature. The non-monotonic behavior of the total Berry curvature is also captured. Increasing the temperature to $T = 300$ K – which is on the order of the of band gap – the normalized CD-RIXS signal is reduced. However, the qualitative behavior reflecting the Berry curvature is clearly visible even at room temperature.


[1] D. Sangalli, E. Perfetto, G. Stefanucci, and A. Marini, An ab-initio approach to describe coherent and non-coherent exciton dynamics, Eur. Phys. J. B **91**, 171 (2018).

[2] E. Perfetto, D. Sangalli, A. Marini, and G. Stefanucci, First-principles approach to excitons in time-resolved and angle-resolved photoemission spectra, Phys. Rev. B **94**, 245303 (2016).

[3] K. Gilmore, J. Pelliciari, Y. Huang, J. J. Kas, M. Dantz, V. N. Strocov, S. Kasahara, Y. Matsuda, T. Das, T. Shibauchi, and T. Schmitt, Description of Resonant Inelastic X-Ray Scattering in Correlated Metals, Phys. Rev. X **11**, 031013 (2021).

[4] Y. Murakami, M. Schüler, S. Takayoshi, and P. Werner, Ultrafast nonequilibrium evolution of excitonic modes in semiconductors, Phys. Rev. B **101**, 035203 (2020).

[5] X. Qian, J. Liu, L. Fu, and J. Li, Quantum spin Hall effect in two-dimensional transition metal dichalcogenides, Science **346**, 1344 (2014).

[6] S. Wu, V. Fatemi, Q. D. Gibson, K. Watanabe, T. Taniguchi, R. J. Cava, and P. Jarillo-Herrero, Observation of the quantum spin Hall effect up to 100 kelvin in a monolayer crystal, Science **359**, 76 (2018).

[7] S. Tang, C. Zhang, D. Wong, Z. Pedramrazi, H.-Z. Tsai, C. Jia, B. Moritz, M. Claassen, H. Ryu, S. Kahn, J. Jiang, H. Yan, M. Hashimoto, D. Lu, R. G. Moore, C.-C. Hwang, C. Hwang, Z. Hussain, Y. Chen, M. M. Ugeda, Z. Liu, X. Xie, T. P. Devereaux, M. F. Crommie, S.-K. Mo, and Z.-X. Shen, Quantum spin Hall state in monolayer 1T'-WTe$_2$, Nature Phys. **13**, 683 (2017).

[8] A. Marrazzo, M. Gibertini, D. Campi, N. Mounet, and N. Marzari, Relative Abundance of Z2 Topological Order in Exfoliable Two-Dimensional Insulators, Nano Lett. **19**, 8431 (2019).

[9] S.-Y. Xu, Q. Ma, H. Shen, V. Fatemi, S. Wu, T.-R. Chang, G. Chang, A. M. M. Valdivia, C.-K. Chan, Q. D. Gibson, J. Zhou, Z. Liu, K. Watanabe, T. Taniguchi, H. Lin, R. J. Cava, L. Fu, N. Gedik, and P. Jarillo-Herrero, Electrically switchable Berry curvature dipole in the monolayer topological insulator WTe 2, Nature Phys. **14**, 900 (2018).
